# Research into the Influence of Volume Fraction on the Bending Properties of Selected Thermoplastic Cellular Structures from a Mechanical and Energy Absorption Perspective

**DOI:** 10.3390/polym17202795

**Published:** 2025-10-19

**Authors:** Katarina Monkova, Peter Pavol Monka, Damir Godec, Monika Torokova

**Affiliations:** 1 Faculty of Manufacturing Technologies with a seat in Presov, Technical University of Kosice, Sturova 31, 080 01 Presov, Slovakia; peter.pavol.monka@tuke.sk (P.P.M.); monika.torokova@tuke.sk (M.T.); 2 Faculty of Technology, Tomas Bata University in Zlin, Vavreckova 5669, 760 01 Zlin, Czech Republic; 3 Faculty of Mechanical Engineering and Naval Architecture, University of Zagreb, HR-10000 Zagreb, Croatia; damir.godec@fsb.unizg.hr

**Keywords:** bending, energy, cellular structures, additive manufacturing, thermoplastic, mechanical properties

## Abstract

The aim of the manuscript is to study the effect of volume fraction on the bending properties of selected thermoplastic cellular structures (Primitive, Diamond, and Gyroid) from a mechanical and energy absorption perspective, with a view to their promising prospects and use not only for bumpers, but also for various vehicle and aircraft components, or other applications. Samples belonging to the group of so-called complex structures with Triply Periodic Minimal Surfaces, dimensions of 20 × 20 × 250 mm, and volume fractions of 30, 35, 40, 45, and 55%, were prepared by PTC Creo 10.0 software and produced using the Fused Filament Fabrication technique from Nylon CF12 material, while the basic cell size of 10 × 10 × 10 mm was maintained for all samples and the volume fraction was controlled by the wall thickness of the structure. Experimental bending tests were performed on a Zwick 1456 machine and based on recorded data; in addition to the maximum forces, the stiffness, yield strength, and effective modulus of elasticity in bending were evaluated for individual structures and volume fractions. Furthermore, the amount of energy absorbed until reaching the maximum force and until failure was compared, as well as the ductility indices *μ*_d_ and *μ_U_* (derived from deformation and absorbed energy, respectively), as an important dissipation factor in absorbers, based on which it is also possible to predict which of the structures will have better damping.

## 1. Introduction

The development of science and technology affects the progress of various sectors and industries. It allows components to be produced faster, more efficiently, and more reliably. Not only new technologies and materials, but also computer-aided design and simulations play a key role. Technologies that are advancing by leaps and bounds today include 3D printing, which reduces the consumption of materials for the fabrication of a component, since the processes for removing unnecessary material from the semi-finished product that were necessary in conventional production are no longer needed [1,2].

The main advantage of 3D printing is the possibility of manufacturing a part with a complex shape that cannot be manufactured conventionally. A special area of components is the so-called lightweight components with regularly spaced structures inside the component body. From a certain point of view, this lightweight material is a three-dimensional (3D) reticulated “foam” with a special structure, but the pore support is connected and oriented according to certain rules in porous materials. This means that the pores are not randomly shaped and distributed, so their properties are better controlled [3,4,5].

Porous materials can give certain products specific exceptional properties. In addition to making the component lighter and having exceptional mechanical properties per unit volume of material, they can also absorb a lot of energy and therefore serve as good “absorbers”. This makes them very promising for application in various industrial sectors, from aerospace through to civil and automotive industries to biomedicine [6].

For example, in cars, bumpers play a very important role in terms of safety. They must be deformable, elastic, absorb impacts, and prevent permanent damage. Car manufacturers use a variety of materials to make bumpers, with aluminum, fiberglass, and composites often used, as well as thermoplastic materials [7,8].

To compare the quasi-static bending from an energetic point of view, the authors selected three types of structures, so-called Primitive, Diamond, and Gyroid, made of thermoplastic Nylon CF12, as a promising perspective for their use not only for bumpers, but also for other components of vehicles, aircrafts, or for other applications.

## 2. State of the Art

In recent years, intensive research has been conducted worldwide on the various properties of cellular structures and their application possibilities. There are also several studies specifically focused on the mechanical properties of thermoplastic materials and porous structures evaluated from an energy point of view. These include, for example, the following studies.

Bernard [9] hypothesized that cellular solids absorb energy more efficiently than monolithic materials. Lattice materials are particularly valuable for their predictable behavior and adaptability through additive manufacturing. His study investigated the strength of multi-layer multi-topology lattices (MLMTs) made from nylon and carbon fiber composites. The material properties were tuned by the orientation of the struts, and single- and three-layer cubic and octet lattices were compared at 30% relative density. The results show that the unique MLMT designs do not exceed the energy absorption of their combined layers, but that modifying the layer interfaces can improve the results. The cubic-octet-cubic MLMT lattice outperformed the octet lattice in mass-specific energy absorption, while the octet-cubic-octet lattice improved the plateau voltage over its MLMT counterpart, although the cubic lattices had higher absorption overall.

The study of Sahu [10] examined how honeycomb cell size affects compressive strength, flexural strength, beam deflection, and impacts behavior. TPE nylon cores showed both in-plane cushioning and out-of-plane energy absorption. Smaller cells (<15 mm) failed by crushing but absorbed more energy, while 15–36 mm cells offered the best in-plane cushioning with lower flexural strength. Larger cells reduced overall beam deflection due to plastic buckling. Drop impact tests confirmed that smaller cells (10 mm) absorbed more energy, which decreased with larger cell sizes. Experimental results matched FEM simulations. Cells under 15 mm are ideal for energy absorption, while 15–36 mm cells excel at cushioning. TPE filament’s flexibility makes it suitable for use as a backing layer in bulletproof vests, helping absorb energy and reduce blunt trauma.

The bending performance of 3D-printed lattice beams, focusing on octet and cube structures, was examined by Yalçın [11] Results showed that energy absorption, specific energy absorption (SEA), and specific load capacity (SLC) depend on lattice type, layer count, and arrangement. Octet beams consistently outperformed cube beams; for instance, in a triple-layer double column set up, the octet beam absorbed 38% more energy and had a higher SEA (0.39 J/g vs. 0.29 J/g). Force–displacement analysis revealed that the octet specimen also supported 43% greater maximum load. While mixed-lattice beams like octet-cube-octet showed benefits, uniform octet lattices achieved the best energy absorption and load capacity. The study also identified two deformation regions—stretch-dominated and compressive-dominated—where vertical struts contributed less to load-bearing in the former, explaining the lower capacity of cube beams compared to octet ones. However, vertical struts were more effective in compressive-dominated regions.

Calignano [12] investigated mechanical properties of components fabricated with a low-cost printer utilizing carbon fiber-reinforced nylon filament. The results showed that components produced in the XY plane exhibit higher hardness than those manufactured in the XZ plane. Within the XZ orientation, samples with 80% and 100% infill demonstrate similar hardness; however, at 100% infill, the hardness of XZ plane specimens is substantially lower than that of their XY counterparts. Specimens printed along the XZ axis are stiffer than those printed along the XY axis, as evidenced by higher Young’s modulus and stress at break, while energy at break is greater for samples from the XY plane. As previously noted, the relationship between mechanical properties and filing factors remains nonlinear.

Additively manufactured (AM) nylon matrix composites reinforced with short and continuous carbon fibers under tensile loading were tested by Islam and his team [13]. It also compares how fiber volume fraction affects mechanical properties using both experimental results and literature. Tensile tests show that nylon with 5% continuous carbon fiber achieves higher tensile strength and Young’s modulus, but lower elongation at break than nylon with 20% short carbon fiber, due to the advantages of continuous fibers. Fracture surfaces indicate brittle failure and fiber pull-outs in the continuous fiber composite, suggesting weak matrix–fiber bonding. Mechanical property trends are presented for varying fiber content, showing a near-linear increase in strength and stiffness for continuous fibers at high volume fractions, while short fibers at low content display no clear relationship.

Monkova [14] investigated how loading and layering directions affect the flexural properties of 3D-printed Nylon CF12, with implications for lightweight sports equipment like pole vault poles. Full-volume and porous samples (Diamond, Primitive, Gyroid) at fixed volume fractions of 30% underwent three-point bending tests. Results revealed that full-volume beams’ flexural properties depend heavily on loading direction and printing strategy, whereas porous cellular beams show minimal differences. At the given volume fraction, the Diamond structure exhibited the highest yield strength among porous designs, while Gyroid had the lowest. Layer adhesion and material deposition paths likely influence these outcomes.

The article of Alfouneh [15] has presented a topology optimization approach for multi-layer, multi-material composite structures under static loads. The method extends moving iso-surface threshold optimization with a physical response function discrepancy scheme and integrates an alternating active-phase algorithm. Three objective functions are addressed: minimizing compliance, maximizing mutual strain energy, and minimizing full-stress (von Mises stress) designs. Benchmark examples demonstrate that the approach yields effective, stiffer, and highly optimized layouts compared to the existing literature, and using multiple materials in layered plates can enhance compliant mechanism performance. All objectives show smooth convergence during optimization, and fully stressed designs have layouts similar to those minimizing compliance.

Xie and Shen [16] investigated the effect of hole shape, size, and distribution on the impact response of porous materials by comparing a spherical hole array model and a square hole model using both an experimental approach and a finite element method. They found that the specific energy absorption capacity correlated with the hole shape, size, and deformation mode, and that, unlike spherical holes, the deformation in square holes was always caused by compression dominance, either with open or closed cells.

There are certainly several other studies that dealt with the mechanical properties of some cellular materials [17,18,19,20,21,22,23], as well as a small number of studies that compare energy absorption between structures [24,25,26,27], but a study that would investigate the effect of volume fraction on the bending properties of complex structures of the Gyroid, Primitive, and Diamond type made of Nylon CF12, and at the same time compare their behavior from a mechanical and energetic point of view as presented in this research, has so far been absent. The obtained results can contribute to the expansion of knowledge not only in the field of material behavior of the thermoplastic Nylon CF12 during bending, but also in the field of investigating cellular structures from an energetic point of view, given their prospective application in many industrial and consumer sectors.

## 3. Materials and Methods

### 3.1. The Samples Design and Production

**Figure 1:**
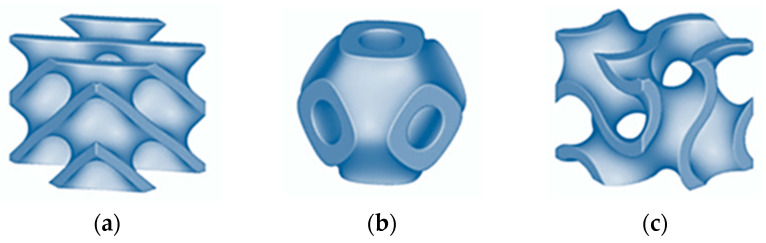
Basic cells of the investigated structures: (**a**) Diamond; (**b**) Primitive; (**c**) Gyroid.

To investigate bending properties from energetic point of view, three types of cellular structures were used—Diamond, Primitive and Gyroid—which belong to the category of Triply Periodic Minimal Surfaces (TPMSs). They are based on three-dimensional surfaces that minimize surface area for a given boundary. Additionally, they are triply periodic, repeating in three independent spatial directions, resulting in crystallographic symmetry. They have a zero-mean curvature at every point, indicating that the surfaces are locally flat and lack sharp edges [28]. The basic unit cells of the above structures designed in PTC Creo 10.0 software, similarly to the whole samples, are shown in Figure 1.

The selection of the structures, as well as the Nylon CF12 material, was based not only on their promising energy absorption properties and possible applications in the automotive and aerospace industries, but also on ongoing research into the behavior of cellular structures, which has been conducted at the authors’ home institution for several years, while the technological conditions of 3D printing were also optimized.

Nylon CF12 is a carbon-filled thermoplastic, featuring 35% short carbon fibers by weight in a Nylon 12 matrix. It offers superior flexural strength and stiffness-to-weight ratio among FDM thermoplastics. Ideal for strong yet lightweight tooling, functional prototypes, and end-use parts in aerospace, automotive, and industrial sectors, it resists heavy mechanical stress and harsh conditions while maintaining structural integrity. Its stability, strength, lightness, and vibration resistance also make it suitable, e.g., for drone frames or components requiring damping properties [29,30].

Physical properties of Nylon CF12 are listed in Table 1.

The material in the form of a filament with a diameter of 1.75 mm was delivered wound on a spool and vacuum-packed, suitable for direct use without the need for drying, and its properties were verified in ongoing tests, confirming the specifications given in the material sheet.

Given the manufacturability of the 10 × 10 × 10 mm basic cell of all selected structures from Nylon CF12, established based on previous research [31], a minimum material volume fraction (defined as the ratio of the volume of the material required to create the structure inside the cell to the total volume of the cell space [32]) of *Vr* = 30% was chosen to determine the effect of volume fraction on bending properties. Subsequent volume fractions of 35, 40, 45, and 55% were established to examine their respective effects, with the highest fraction selected to ensure a discernible reduction in the weight of the final products, while the volume fraction was controlled by the wall thickness of the structure.

Three identical samples were produced from each series specified by volume fraction and structure type, so a total of 45 samples were manufactured (3 structures × 5 volume fractions × 3 pieces). Samples’ total sizes of 20 × 20 × 250 mm (2 × 2 × 25 cells) were printed using the Fused Filament Fabrication (FFF) technique employing a MarkerBot Method X printer (MakerBot, New York, NY, USA) with MarkerBot Cloud software 3.10.1 with the following production parameters set up based on preliminary research [33]: filament diameter, 1.75 mm; nozzle diameter, 0.4 mm; temperature in the printer chamber, 65 °C; extruder temperature, 260 °C; printing speed, 35 mm/s; travel speed, 80 mm/s; layer height, 0.15 mm; bed temperature, 65 °C; printing technique, stripes with 45° degree angle and 90° degree rotation for every second layer.

**Figure 2:**
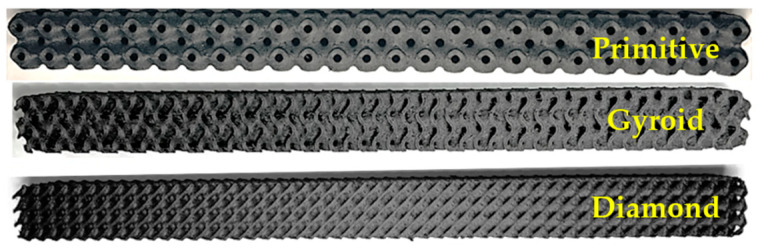
Example of produced samples with 35% volume fraction.

An example of produced samples with 35% volume fraction is in Figure 2.

### 3.2. Testing and Evaluation Methodology

**Figure 3:**
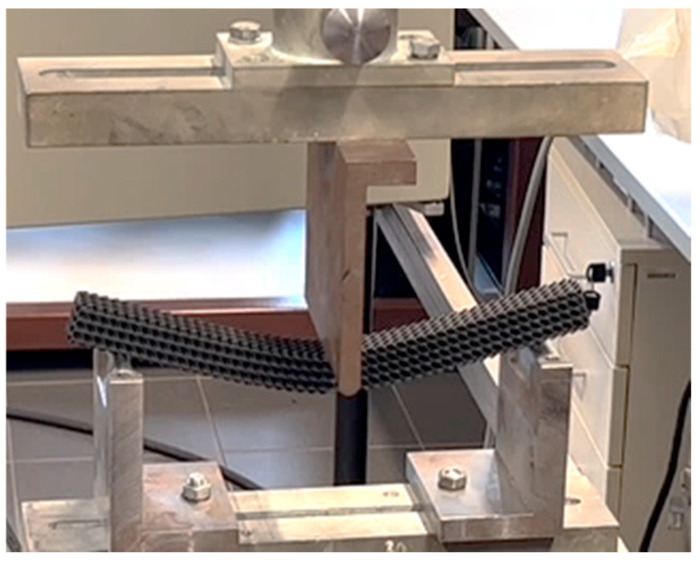
Sample during testing when placed in the experimental set up of the testing machine.

Experimental bending tests were performed on a Zwick 1456 machine (Ulm, Germany) according to ISO 178:2019 [34] standard at a temperature of 20 °C and a humidity of 50%. The support span distance of *l* = 200 mm was set symmetrically around the center of the specimen; the pressure thumb had a radius of 10 mm and operated at a crosshead loading rate of 10 mm/min. The experimental set up is shown in Figure 3.

The crosshead speed of 10 mm/min was set up based on the standards of ASTM D790 (3-point flexural test for plastics) [35] and ISO 178 (plastics—determination of flexural properties) [34].

ASTM D790 has Procedure A (for materials that break at comparatively small deflections, it uses a strain rate of 0.01 mm/mm/min) and Procedure B (for materials that yield large deflections, it uses 0.10 mm/mm/min). ASTM D790 gives the formula for crosshead speed (displacement rate) calculation. For the material Carbon fiber-reinforced Nylon (i.e., thermoplastic composite, moderately stiff and brittle) used in our case, with a support span of 200 mm and cross-section (width × depth) of 20 mm × 20 mm, the target crosshead speed was calculated based on the standard recommendation as 66.7 mm/min. This keeps strain rate at 0.01 mm/mm/min, matching quasi-static requirements.

ISO 178 recommends 1 %/min strain rate for rigid plastics that also leads to a crosshead speed of 66.7 mm/min. So, both the ASTM D790 and ISO 178 standards are essentially the same in the crossbar speed prescription.

For 70% porosity (*Vf* = 30%), the effective modulus is reduced which means that material stiffness behaves much more flexibly, so calculated crosshead speed was close to 30 mm/min. The authors decided to use a crosshead speed of 10 mm/min, which is much slower than the standards prescribe, to be sure that they are in the quasi-static loading region.

**Figure 4:**
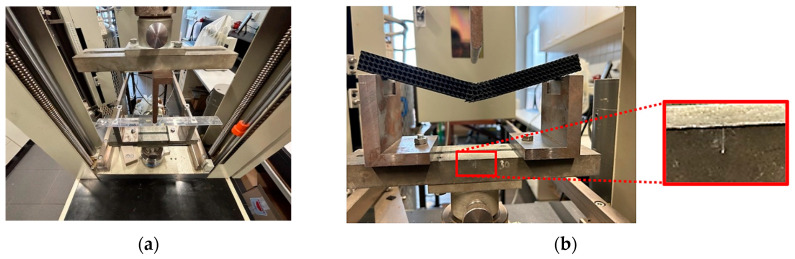
Central position of a sample checking (**a**) before experiments were run; (**b**) aligning the marked center of the sample with the center of the push thorn.

The central position of the pressure finger relative to the supports was ensured by the test machine fixtures (checked before the experiments were run, Figure 4a), and the symmetrical position of the sample relative to the push thorn was checked before each experiment by aligning the marked center of the sample with the center of the push thorn, as well as with respect to the marked center between the supports (Figure 4b).

Outliers from further measurements and calculations were excluded by statistical processing, while the recorded differences in results between identical samples within one series were in all cases less than 5%.

Several outcomes were assessed using recorded data and load–deflection curves based on the fundamental theory of the three-point bending test.

**Figure 5:**
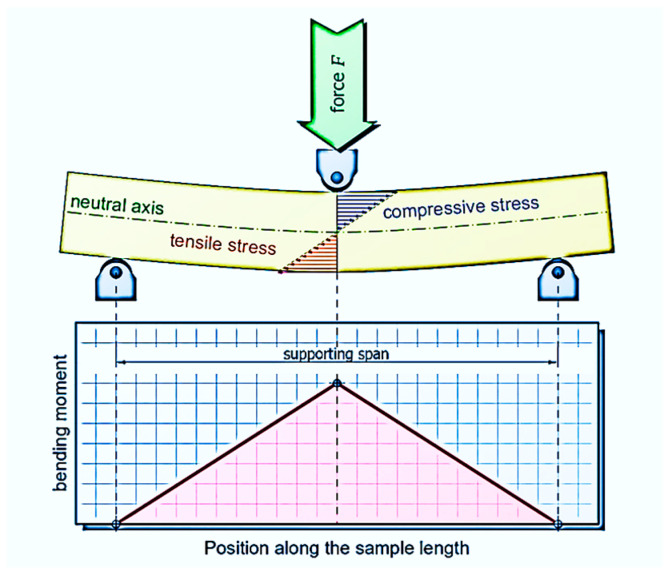
Three-point bending test in theory—bending moment distribution.

The bending load has its highest value in the cross-section of the maximum deflection, i.e., in the middle of the specimen, as is seen in Figure 5. The material is subjected to compressive stress on the inside and tensile stress on the outside side, while the stress values are highest at the surface layer of the material due to the maximum compression or strain. In so-called neutral axis, the tensile and compressive stresses are distributed identically over the cross-section.

Assuming a linear stress distribution, the bending moment *M* relates to the stress *σ* (Pa) at a distance “*z*” from the neutral axis, based on the specimen’s cross-sectional geometry defined by the area moment of inertia *I* (also referred to as the second moment of area) [36].

(1) $$\sigma =\frac{M}{I}z\;,$$

The limit to which the materials can be stressed at a given bending load without permanent deformations is called flexural yield strength. The induced bending moment *M_y_* (Nm) at the onset of plastic deformation can be determined with the yield force *F_y_* according to the equation

(2) $$M_{y}=\frac{F_{y}l}{4}\;,$$

where *l* is a supporting span.

During loading, the energy is stored in the structure. It can be said that a structure’s energy is its capacity to do work. In linear elasticity, the entire deformation work is converted into elastic tension energy and strain energy is the internal energy in the structure because of its deformation [37]. By the principle of conservation of energy,

*Wi* = *U* (3)

where *U* denotes the strain energy and *Wi* represents the work done by internal forces. For linearly elastic structures, the partial derivative of the strain energy concerning an applied force *F* is equal to the displacement *u* of the force along its line of action [38,39]:

(4) $$u=\frac{\partial U}{\partial F}$$

The strain energy of a beam can be expressed by the equation [40]:

(5) $$U=\int_{0}^{l}\frac{M^{2}}{2EI}dx.$$

where *E* is flexural elastic modulus (Pa) of the beam material. Finding the partial derivative of this expression will give the equations of Castigliano’s deflection and rotation of beams.

According to elementary beam theory, the applied bending moment *M* is given as follows [41]:

(6) $$M=EI\frac{d^{2}w}{dx^{2}},$$

where *x* is the distance along the beam. Then, double integration of Equation (6) results in the beam’s deflection.

Based on the energy approach, the flexural modulus of elasticity *E* can also be determined. If the elastic deflection *w_e_* (m) is expressed by Equation (7)

(7) $$w_{e}=\frac{F_{e}l^{3}}{48EI},$$

then flexural elastic modulus can be expressed

(8) $$E=\frac{F_{e}l^{3}}{48w_{e}I},$$

where *F_e_* denotes the applied elastic force (N).

It becomes apparent from Equation (7) that the greater the product of flexural modulus *E* (material property) and area moment of inertia *I* (geometric property), the less the specimen deflects. For this reason, this product *EI* is often referred to as flexural rigidity *R_f_* (Nm^2^) which refers to the ability of a material to maintain its shape under stress or load, while bending stiffness *k_b_* (N/m) refers to the ability of a material to resist deformation when subjected to an external load, and it comes from the relation [42]

(9) $$k_{b}=\frac{F_{e}}{w_{e}}$$

The sample can continue to be plastically deformed with tough materials as the load increases. Compared to ductile materials, brittle ones break without any noticeable deformation [43].

When evaluating results from an energetic point of view, total energy absorption can be found by integrating the force–deflection curve. Subsequently, based on energy, the ductility of the beams can also be accessed through two indices, *µ_d_* and *µ_U_*, that are given by Formulas (9) and (10) [44,45]:

(10) $$\mu_{d}=\;\frac{w_{u}\;}{w_{e}}$$

(11) $$\mu_{U}=\;\frac{1}{2}\left(\frac{U_{tot}\;}{U_{e}}+1\right)$$

where *w_u_* (mm) is the deflection at the ultimate load and *w_u_* (mm) is the deflection at the elastic limit; *U_tot_* (J) is the total energy absorbed by the sample during bending; and *U_e_* (J) is the elastic energy absorbed up to the elastic limit.

## 4. Results and Discussions

### 4.1. Experimental Results

**Figure 6:**
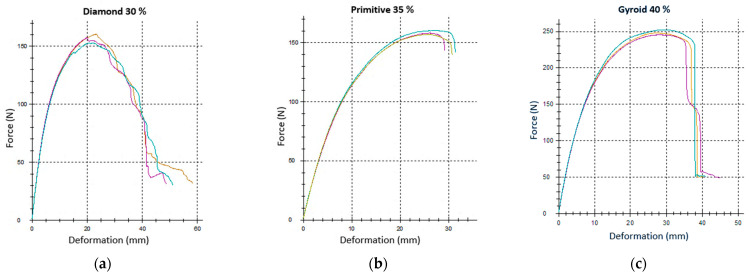
Load–deformation curves recorded during experimental testing for repeated measurements of identical samples within a single series: (**a**) Diamond 30%; (**b**) Primitive 35%; (**c**) Gyroid 40%. (The results of three samples of the same type, tested for repeatability and statistical processing, are inserted into one graph, while the recorded dependencies are shown in the graph with a different line color for clarity).

In order to determine the behavior of samples of Nylon CF12 material with the selected topology characterized by the type of structure and volume fraction while maintaining the cell size of 10 × 10 × 10 mm, experimental testing was carried out using a three-point bend. During testing, curves of the dependence of the applied force on the deformation of the beam were recorded. Examples of recorded dependencies of three identical samples, which were subjected to testing with respect to repeatability and statistical data processing for three different structure topologies, are shown in Figure 6.

As is evident from the displayed dependencies, the samples in each series showed good agreement of the measured data and the reliability of repeated measurements, which was also confirmed by the results of statistical processing focusing on the Grubbs’ test, with only one outlier found, which was excluded from further data processing.

**Figure 7:**
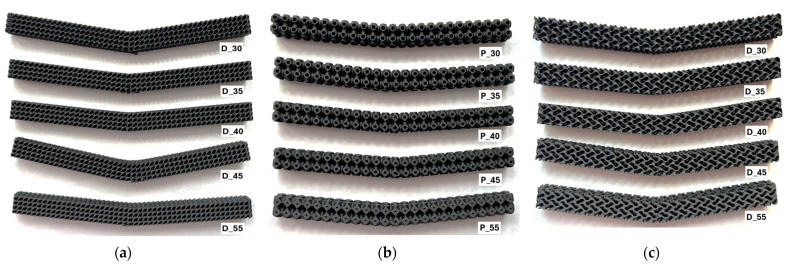
An example of damaged samples with different volume fractions: (**a**) Diamond; (**b**) Primitive; (**c**) Gyroid.

An example of one collection of post-failure samples divided by structure type and arranged in ascending order of volume fraction is shown in Figure 7. According to the assumption, failure occurred at the point of highest stress in the middle of the lower part of the specimens and crack propagation proceeded by breaching the intercellular walls upwards.

**Figure 8:**
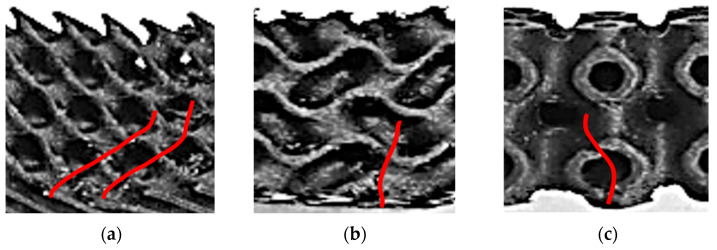
An example of crack propagation (red lines) at the samples with *V_f_* = 30%, magnification 2:1: (**a**) Diamond; (**b**) Gyroid; (**c**) Primitive.

For structures with a volume fraction of 30%, failures occurred approximately in the middle of the beam, around the point of the largest bending moment, but at the locations of the highest stress concentration. In the Diamond structure (*V_f_* = 30%), two crack propagations were observed after failure, which proceeded obliquely upwards perpendicular to the cell walls (Figure 8a); in the Gyroid (Figure 8b), this was similar, but due to the larger pore size, the propagation was steeper. In the Primitive structure (Figure 8c), the deflection of the crack propagation was caused by the perpendicular orientation of the cell wall of the internal pore, which in this case acted as a reinforcing element.

As volume fraction/wall thickness increased, a shift could be seen as slender elements that could previously buckle or bend plastically were at some stages comparatively rigid; the load carried by the walls was higher and local stresses likely reached levels that caused fracture instead of allowing stable plastic deformation. Also, increased wall thickness reduced slenderness; increased stiffness reduced the amplitude of buckling but increased stress concentration at defects, etc.

The threshold volume fraction (or relative density), at which the failure mechanism transitions (from buckling/ductile to brittle), depends not only on the geometry (topology), but also on the material and the quality (defects, etc.). For our observed samples, those volume fractions, 40% for Gyroid and 45% for Diamond, are plausible thresholds, which are related to the material Nylon CF12 and print/processing conditions.

**Figure 9:**
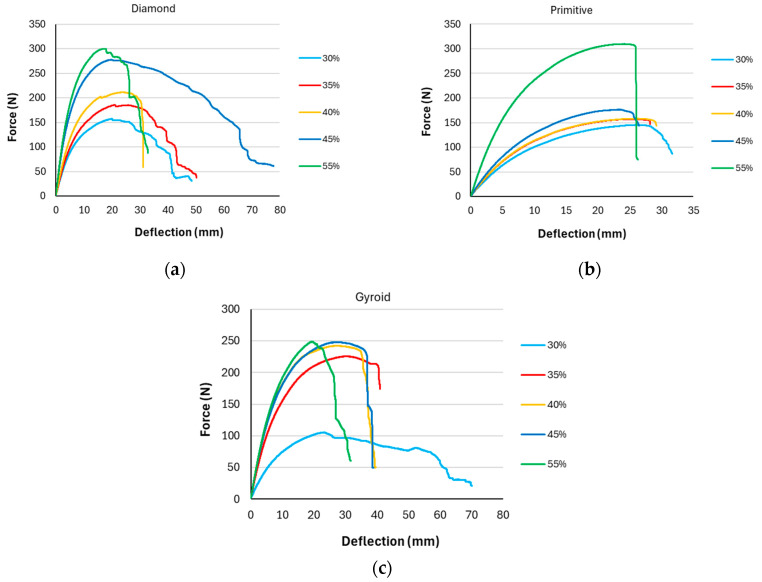
Representative force–deflection curves with different volume fraction for individual structures: (**a**) Diamond; (**b**) Primitive; (**c**) Gyroid.

To assess the influence of volume fraction *V_f_* on the behavior of samples during bending loading, the measured data were processed and grouped by type of structures. The average values of measured maximal forces are listed in Table 2, while representative curves of one of the series of samples for each structure type are shown in Figure 9.

As expected, with increasing volume fraction, the maximum measured force also increased; however, its increase was not regular regarding the volume fraction, but for different types of structures the behavior was different, and similarly with regard to the deflection achieved at sample failure. Therefore, examining the behavior of structures from various perspectives, including energetic viewpoint, could provide a more comprehensive understanding.

### 4.2. Evaluation of Research Results from Mechanical and Energy Absorption Perspective

When evaluating the measured data and dependencies, at first, the bending stiffness was calculated for each structure topology given by the type of structure and the volume fraction.

**Figure 10:**
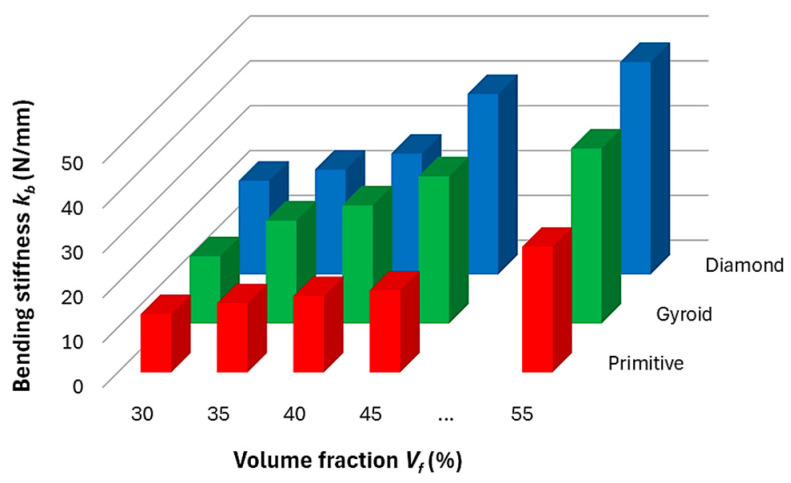
Bending stiffness.

From the histogram in Figure 10, it is clear that stiffness increases with increasing volume fraction, which was expected. However, it is possible to observe different ways of increasing stiffness with increasing volume fraction for different structures. For the Primitive structure, the increase in stiffness is slow, and only at a volume fraction of 55% is a sudden change evident, while the values for the studied area were in the range of 13.2–28.4 N/mm.

For Gyroid, the dependence of *k_b_* on *V_f_* appears to be linearly increasing with values from 15 to 39.3 N/mm for extreme values of volume fraction of 30 and 55%, while the highest stiffness values (20.9–47.3 N/mm) within the investigated range of volume fraction were shown by the Diamond structure and the increase trend is related to an exponential function.

The irregular growth of stiffness occurs because cellular materials have a nonlinear response as they approach a more rigid mass-like structure. This transition depends on the architecture of the unit cells and the mechanical properties of the material used. It can be said that as the volume fraction in a cellular structure increases, the relative thickness of the cell walls also increases, which affects the stiffness due to the bending and stretching of the material in the structure. Thicker walls contribute more to the overall stiffness, while thinner walls make the structure more flexible, as described in the studies [46,47]. Cellular materials are also prone to stress concentrations at the intersections of cell walls and pores, which act as notches [48]. Higher volume fraction can lead to a transition where the material becomes less porous and more rigid, which affects both stress transfers.

**Figure 11:**
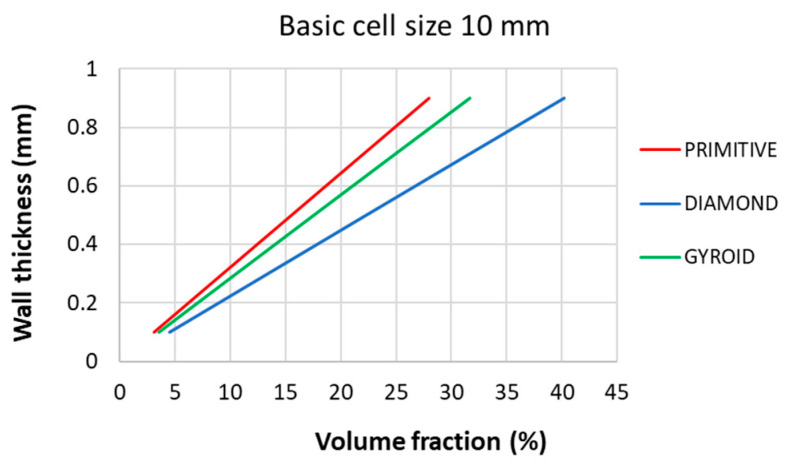
Dependencies of wall thickness on volume fraction for all three studied structures with a basic cell size of *a* = 10 mm.

The authors therefore made a quick overview of the dependence of the wall thickness of the structure on the volume fraction, and found that the differences between the wall thicknesses of the structure are relatively large for the same volume fraction of the samples. Below, in Figure 11, the authors generated dependence of the wall thickness on the volume fraction for all three types of structures with a basic cell size of *a* = 10 mm.

It is possible to obtain wall thickness values for higher volume fractions based on the trend of the dependencies given by the equations (with coefficient R^2^ = 1 in all three cases):

Diamond:

*y* = 0.0224*x* + 0.001 (12)

Gyroid:

*y* = 0.0284*x* + 0.0012 (13)

Primitive:

*y* = 0.0322*x* + 0.0001 (14)

The graph in Figure 11 explains why the Primitive structure exhibits the lowest mechanical properties and the Diamond structure the highest, and demonstrates that wall thickness is a significant factor influencing the properties of the structures if the volume fraction (i.e., the amount of solid phase material used in the production of samples) is set as a criterion for comparing the behavior of the structures.

Analysis of the results suggests that the stiffness mutation observed at 55% volume fraction in the Primitive structure could be due to an increase in cell wall thickness, which causes the material to transition from a “porous support” structure to a “quasi-rigid” structure, where the structure is more continuous and the material can carry a greater load. This transition is likely to lead to a shift in the mechanical properties of the material.

The Gibson-Ashby model [49] describes the mechanical behavior of cellular solids by relating their effective modulus to their relative density (volume fraction). The model predicts the stiffness of the structure based on geometric factors (such as cell wall thickness) and material properties. However, the Gibson-Ashby model assumes isotropic structures, which may not be applicable to more complex, anisotropic lattice structures such as Gyroid or Diamond, which may require more sophisticated models to account for their geometric anisotropy as well as realistic manufacturing conditions and the influence of technological parameters.

**Figure 12:**
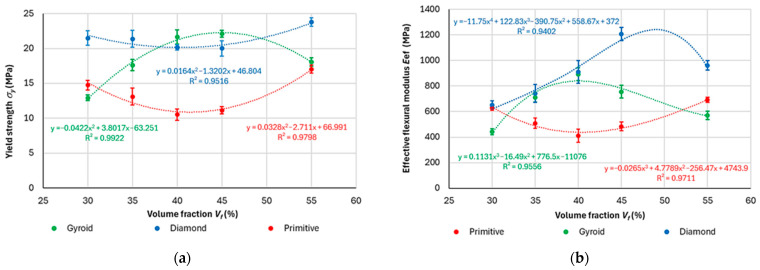
Dependence of: (**a**) yield strength; (**b**) effective flexural modulus of elasticity, on volume fraction.

For practical applications, it is essential to know the behavior of samples within elastic regions to ensure that components function within a safe range, thereby preventing plastic deformation and potential damage. By means of Equations (2) and (8), and entering the geometric characteristics of the structure using software in which the 3D models of the samples were generated, the yield strength *σ_y_* and effective modulus of elasticity *E* were calculated, implementing the average value of the force at the elastic point for a specific sample topology, and these are presented in Figure 12. Since the experiments were not directly aimed at specifying Young’s modulus following the necessary standards, the term “effective modulus of elasticity *E*_ef_” is applied in the manuscript for the purpose of presenting the obtained results, which represent the real behavior of the sample under the given conditions.

The effective elastic modulus in bending varied within the range of investigated volume fraction *V_f_* for individual structures according to the trends described by the following equations:

Diamond: (R^2^ = 0.9516)

*y* = 0.0164*x*^2^ − 1.3202*x* + 46.804 (15)

Gyroid: (R^2^ = 0.9922)

*y* = −0.0422*x*^2^ + 3.8017*x* − 63.251 (16)

Primitive: (R^2^ = 0.9798)

*y* = 0.0328*x*^2^ − 2.711*x* + 66.991 (17)

It is clear that in the investigated volume fraction range of 30–55% (limited by manufacturability and meaningful applicable material lightweighting) there is a region (minimum for Primitive and Diamond structures, respectively; maximum for Gyroid structure) in which the stresses become extreme, which can mean that this combination of material, structure, technological conditions, cell size, skin thickness, and volume fraction (controlled by wall thickness) is the most suitable (respectively, the most unsuitable) for use when considering the behavior of the sample in the elastic region.

With a high coefficient of determination R^2^, which states how well the statistical model predicts the outcomes [50], it is possible to calculate a more accurate volume fraction in which the function takes an extreme. For example, in the Gyroid structure, where a second-order polynomial describes the behavior of the structure in terms of yield strength with respect to volume fraction with relatively high reliability (R^2^ = 0.9922 is high, close to 1), it is possible to determine through computation that the extremum of the function (maximum value of yield strength) will be at a volume fraction of *V_f_* = 45.044%.

Similarly, the dependence functions of the effective modulus of elasticity *E_ef_* on the volume fraction *V_f_* for individual structures were generated, while to describe the dependencies with relatively good reliability of predicting the outputs, it was necessary to use polynomials of a higher degree (third order for Gyroid and Primitive, and fourth order for Diamond). The obtained dependency equations are as follows:

Diamond, R^2^ = 0.9402

*y* = −11.75*x*^4^ + 122.83*x*^3^ − 390.75*x*^2^ + 558.67*x* + 372 (18)

Gyroid, R^2^ = 0.9534

*y* = 0.1139*x*^3^ − 16.604*x*^2^ + 781.62*x* − 11149 (19)

Primitive, R^2^ = 0.9711

y = −0.0265*x*^3^ + 4.7789*x*^2^ − 256.47*x* + 4743.9 (20)

In this case, it also appears that the functions in a given range of volume fractions reach an extreme at which the configuration of input parameters for the effective modulus of elasticity *E*_ef_ (*E*_ef_ = *σ*_y_/ε) is the most (or the least) appropriate. On the basis of the dependencies, it is also inversely possible to determine the necessary volume fraction of the material for the required modulus of elasticity, since sometimes in practice, for low-stressed components, the yield strength values are not the most important, but the strain is more important.

A measure of the energy dissipation capacity of a structure is given by ductility, which is the deformation capacity of a structure after the first yield [51]. Energy dissipation is desirable for structures in many applications since it refers to the loss of energy in a system in quasi-static loading, and materials with high static energy dissipation tend to also have high damping capabilities under oscillatory motion [52].

**Figure 13:**
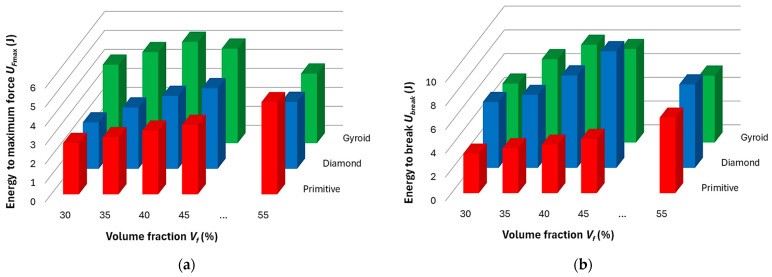
Histograms showing amount of energy (**a**) absorbed up to the maximum force *F*_max_ (N); (**b**) required to break the sample.

**Figure 14:**
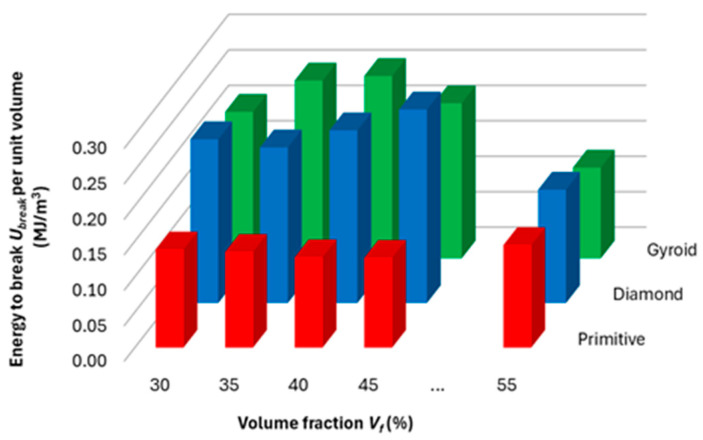
Energy absorption to break per mass unit.

**Figure 15:**
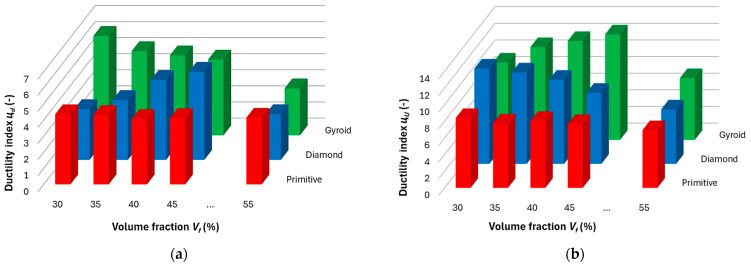
Ductility indices derived from (**a**) deformation and (**b**) absorbed energy for the investigated topologies of the structures.

Based on the methodology presented in Section 3.2, the values of absorbed energy *U_F_*_max_ (J) up to the maximum force *F*_max_ (N), energy required to break the sample *U*_break_ (J), and consequently the values of the ductility indices *μ_d_* and *μ_E_* were calculated for individual samples. The average values achieved from three measurements of identical samples’ topologies are shown in the graphs in Figure 13, Figure 14 and Figure 15, while the deviation of the evaluated variable did not exceed 5% in all cases.

Both histograms in Figure 13 show similar behavior in samples of the same type. In Gyroid and Diamond structures, the values of absorbed energy (both *U_F_*_max_ until reaching *F*_max_ and *U*_break_ until break) increased. Gyroid reached maximum values of absorbed energy at *V_f_* = 40%, unlike the Diamond structure, in which the values were highest at 45% volume fraction, after which the values decreased in both structures. The Primitive structure also showed a gradual increase in absorbed energy with increasing volume fraction, reaching a maximum at the highest volume fraction examined of 55%, but the further development trend (increase or decrease in energy) is difficult to predict from the measured results.

In order to compare the efficiency of material use, and for the purpose of comparing performance with other materials (results of other researchers), the amount of energy absorbed (until the sample breaks) per unit volume of the solid phase was calculated and plotted in the histogram in Figure 14, and the values achieved for energy absorption to break per a unit mass were tabled (Table 3).

It is clear from the graph in Figure 14 that the highest absorbed energy normalized to a unit volume of material is achieved by structures at different volume fractions; while for Gyroid it is at 40% of the Nylon CF12 used and the value of *U*_break_ per unit volume reaches 0.257 MJ/m^3^, for the Diamond structure it is at a volume fraction of *V_f_* = 45%, *U*_break_ per unit volume = 0.273 MJ/m^3^, and for Primitive it is 0.145 MJ/m^3^ at a volume fraction of 55%. Interesting values of material utilization efficiency for evaluating the amount of absorbed energy were shown by the Diamond structure, which at 30% volume fraction reached a value of 0.231 MJ/m^3^, which is higher than the Gyroid structure at *V_f_* = 45%, and than the Diamond structure at *V_f_* = 35%.

After recalculating with the specific gravity of the material (1.19 g/cm^3^) [29], the Diamond and Gyroid structures in our research achieved energy absorption values in the range of 0.174–0.229 J/g, which is in line with the available study by Feng et al. [53], which focused on CFRP tubes filled with bio-inspired stepped lattices and achieved an amount of absorbed energy in the range of 1.33–3.19 J/g. On the other hand, our energy absorption values are about half the results achieved by Ma [54], who dealt with a reinforced star-shaped sandwich beam with auxetic honeycomb (RSSAH) reinforced with hollow thin-walled tubes of the polymer material PLA. Zhou [55] evaluated energy absorption performances of novel auxetic honeycomb circular tubes under different impact loading, made of PLA and designed with a gradient auxetic circular tube (ACT) by adjusting the number of radial honeycomb layers. The amount of energy absorbed reached a value of around 1 J/g for their structures.

In these mentioned cases, the skin covering the structures and its thickness played an important role, as well as the volume fraction of the material and the shape of the samples, which affect the section modulus of inertia to a large extent. The authors of the presented research did not use any skin because they wanted to know the properties of the structures themselves without the influence of the outer skin since their previous research [5] showed that the influence of the outer surface layer of a continuous material (so-called skin) is very large.

Energy absorption performances of hybrid thin-walled tubes with BCC and BCC-Z lattice structures investigated by Cetin [56], which were made of AlSi10Mg, reached 35 J/g under axial impact loading conditions. In this case, the metal material and the method of loading play the most important role.

The ductility indices, *μ_d_* (based on deformation, Figure 15a) and *μ_U_* (based on absorbed energy, Figure 15b), show inverse behavioral trends for individual structures with increasing volume fraction compared to the trend of energy absorption dependences on volume fraction (Figure 13). If the values of one of the indices decrease with increasing volume fraction, the values of the other index increase, while for the Primitive structure the trend is almost constant in both cases.

As mentioned above, in the experimental analysis of structures with Gyroid and Diamond topologies, an inverse relationship was observed between the ductility indices (*μ_d_*, *μ_u_*) and the ability of the material to absorb energy during deformation. With increasing energy absorption, the ductility of the material decreases, which at first glance seems paradoxical, since a higher rate of absorbed energy is often associated with a greater deformation capacity. However, this phenomenon can be explained in terms of the dominant mechanisms of plastic dissipation and the nature of the deformation of the cellular structure itself.

In structures with a higher volume fraction, i.e., denser topologies, more elastic deformation occurred during loading, while the proportion of plastic energy dissipation was limited. The thicker and stiffer cell walls of these structures showed increased resistance to plastic flow and were more prone to elastic buckling or local deformations, which allowed for a more efficient distribution of the loaded energy without permanent damage to the material. This led to a higher value of absorbed energy, but at the cost of reduced ductility, because plastic deformation—and therefore the ability of the material to change its shape without immediate failure—was significantly limited.

In contrast, structures with a lower volume fraction had thinner cell walls, which were more sensitive to plastic mechanisms such as permanent buckling, local fractures, or friction between elements. In such cases, the energy during deformation was absorbed predominantly by plastic work, thereby increasing the ductility of the structure. However, this higher deformation capacity was achieved at the cost of lower overall energy absorption, as the structure was not able to effectively resist sudden or intense loads in an elastic manner alone.

This correlation between ductility, energy absorption, and structural safety has a fundamental impact on the selection of the appropriate topology for a particular application. In cases where impact protection is a priority, such as in automotive deformation zones or protective elements, structures with a higher volume fraction and higher energy absorption are more suitable, even at the cost of lower ductility. Conversely, in applications where high ductility or flexibility is required—such as in biomechanical implants or soft damping components—structures with lower density are more advantageous, allowing for greater plastic deformations. In cases where a compromise between energy absorption and ductility is needed, such as in vibration dampers or multipurpose technical components, solutions with structural gradation, or a combination of different topologies or materials, appear promising.

It follows from the above that the inverse relationship between energy absorption and ductility is not just an empirical observation, but reflects deeper connections between microstructural geometry, deformation mechanisms, and energy dissipation. In the future, when designing functional structures from advanced topologies such as Gyroid and Diamond, it will be therefore essential for the authors to consider not only their static strength, but also their dynamic and plastic behavior in terms of the requirements of the given application.

In connection with the tested material, questions arise regarding its limits in use for specific applications. One of the most important environmental factors that can affect the performance of Nylon CF12 is temperature. Since Nylon, as a synthetic fiber-based polymer, is known for its susceptibility to change in mechanical properties at elevated temperatures, in real practice, where materials are exposed to high temperatures, its thermal degradation can occur. High temperatures can lead to softening of the material, a decrease in its strength and elasticity, and thus a decrease in its ability to withstand mechanical loads [57]. This effect can be particularly critical in applications where the requirements for long-term stability of the material at operating temperatures are high. On the other hand, low temperatures can also affect the performance of the material, especially if Nylon CF12 operates in aerospace applications or in places with extreme winter conditions. At temperatures below freezing, the material can become more brittle and less able to withstand shock or impact loads, which could lead to structural failure [58]. These temperature changes can have a negative impact on the overall service life of components made from this material.

Another factor to consider when selecting Nylon CF12 for a given application is the effect of humidity. Nylon, as a hygroscopic material, tends to absorb moisture from the surrounding environment. In the case of high humid conditions (e.g., in humid environments or when the material is exposed to rain), mechanical properties can degrade, such as a decrease in strength and elastic modulus. Humidity causes water molecules to bind to the polymer chains, which can lead to their swelling and a decrease in intermolecular forces between the chains [59]. This process can ultimately affect not only the strength of the material, but also its resistance to wear and fatigue damage.

Possible improvements in the material properties include, for example, modifying the material mixture to increase moisture resistance and improve stability at high temperatures, or implementing protective coatings or composite materials to provide additional protection against external influences.

For later practical applications, it will be important to find a compromise (using multi-criteria optimization) between the performance characteristics of the sample and the material consumption, while of course it will be necessary to take into account other safety and reliability factors.

## 5. Conclusions

Porous materials can be an effective tool for saving material and reducing the weight of a component while maintaining its desired properties. The aim of the presented research was to determine the effect of volume fraction on the properties of Nylon CF12 in terms of mechanical properties and energy absorption to assess their dissipative properties and damping potential. At the same time, the behavior of three types of complex porous structures (Diamond, Gyroid, and Primitive) was compared when changing the volume fraction, while the same basic cell size was maintained for all samples. The most important findings of the research are as follows:

- The stiffness of the structure increased with increasing volume fraction, and the highest stiffness was shown by the Diamond structure with values from 20.9 to 47.3 N/mm, followed by Gyroid in the range of 15–39.3 N/mm, and finally by the Primitive structure with values of 13.2–28.4 N/mm in the investigated volume fraction range of 30–55%.
- When assessing the behavior of the sample in the elastic region, the results of yield strength and effective elastic modulus showed that within the investigated volume fraction range of 30–55%, there is an area in which the individual structures show the best/worst properties, which may mean that this specific combination of material, structure, technological conditions, cell size, and volume fraction is the most suitable (or least suitable). The dependencies of the above variables on *V_f_* can be described by polynomial functions with relatively good reliability, but the resulting equations and coefficients will need to be verified in the future by testing with a larger number of samples.
- Thicker and stiffer cell walls of these structures have shown increased resistance to plastic flow and are more susceptible to elastic buckling or local deformations, which allow for a more efficient distribution of the loaded energy without permanent damage to the material. This leads to a higher value of absorbed energy, but at the cost of reduced ductility, since plastic deformation—and therefore the ability of the material to change its shape without immediate failure—is significantly limited.
- Until reaching maximum force, the Gyroid structure is best able to absorb energy, and in terms of the total energy that the samples were able to absorb until failure, the Gyroid and Diamond structures were comparable; however, the highest values of absorbed energy were achieved by the Gyroid structure at 40% volume fraction, and the Diamond structure at *V_f_* = 45%.
- The inverse relationship between energy absorption and ductility reflects deeper connections between microstructural geometry, deformation mechanisms, and energy dissipation.
- In terms of ductility, the structures show different behavior. In the area up to the maximum load, the ductility index *μ_d_* based on deflection shows for the Gyroid structure a decreasing character with increasing volume fraction, and, conversely, for the Diamond structure it increases, while at *V_f_* = 55% a significant decrease was already recorded at the Diamond structure.
  In the area of the behavior of the samples up to failure, the ductility index derived on the basis of energy has the opposite trend; for the Gyroid it increases with volume fraction and for the Diamond it decreases.
  The Primitive structure shows stable values with the smallest influence of volume fraction in both ductility indices.
- Based on the research, it can be stated that both Gyroid and Diamond structures appear to be suitable static energy dissipators and the choice of structure will depend on the application and the area in which energy absorption is required (i.e., whether it is a stress area up to maximum load or to total failure). The damping properties during oscillation or impact will be verified and compared in the authors’ research in the near future.

## Figures and Tables

**Table 1:** Physical properties of Nylon CF12 [29].

Property	Unit	Value
Density	g/cm^3^	1.19
Melting point	°C	180–190
Tensile modulus of elasticity *	GPa	3–9.46
Ultimate tensile strength *	MPa	32.7–83.5
Elongation at break	%	1.2–2.4
Toughness	J/m^2^	50

* Depends on printing orientation.

**Table 2:** Measured maximal forces *F*_max_ (N).

	*V_f_*	Structure Type		
		Diamond	Primitive	Gyroid
Maximal Force *F*_max_ (N)	30%	163 ± 6.67	141 ± 5.33	110 ± 4.67
	35%	190 ± 2.67	151 ± 4.67	227 ± 2.33
	40%	213 ± 2.00	158 ± 5.33	242 ± 4.67
	45%	279 ± 4.67	179 ± 4.00	252 ± 6.67
	55%	317 ± 6.67	314 ± 6.33	283 ± 0.50

**Table 3:** Average values of energy absorption to break per unit mass (J/g) for individual structures.

Structure Type	Volume Fraction *V_f_* (%)				
	30	35	40	45	55
Gyroid	0.1737	0.2107	0.2161	0.1837	0.1075
Diamond	0.1940	0.1843	0.2046	0.2292	0.1343
Primitive	0.1162	0.1134	0.1071	0.1064	0.1215

## Data Availability

The data are contained within the article.
